# Conditioned contextual fear memory to assess natural forgetting and cognitive enhancement in rats

**DOI:** 10.14440/jbm.2018.256

**Published:** 2018-09-20

**Authors:** Sven RM Schuette, Scott Hobson

**Affiliations:** CNS Research, Boehringer Ingelheim Pharma GmbH & Co. KG, 88397 Biberach a.d. Riss, Germany

**Keywords:** contextual fear conditioning, memory enhancement, memory acquisition, hippocampus

## Abstract

Aversively established contextual fear memory manifests itself in robust freezing behavior, often lasting several weeks or months. Therefore, this approach is amenable to investigate the underlying neural circuitries by lesion or inactivation of specific brain regions or to test efficacy of substances that disrupt either the ability to acquire the association or to retrieve memories. In contrast, investigation of memory enhancement using this technique is time intensive since the non-treated control group naturally forgets the learned association only weeks after acquisition. Pharmacological interventions have been used to overcome this time span by disrupting memory at any time point, however, limiting it a mechanistic model of reversal of impairments instead of studying memory enhancement. Here, we investigated several parameters of the cued and contextual fear conditioning (CFC) protocol such that, while memory acquisition is established, loss of fear association occurs within a shorter time frame, allowing studies of memory enhancement in the context of natural forgetting. We found that three predictive tone-cues, each separated from a 0.3 mA foot shock by an interstimulus interval of 2 s and a pre-exposure to the context enables the investigation of enhanced contextual memory 7 d post training without the necessity of inducing pharmacological lesions.

## INTRODUCTION

The cued and contextual fear conditioning (CFC) task is a classical pavlovian model to investigate associative memory for the pairing of both a neutral conditioned stimulus (CS, here a tone-cue) and the context with an aversive, unconditioned stimulus (US, here an electric foot shock). Once this pairing is acquired, presenting either the context or the tone-cue alone leads to the conditioned reaction (CR), in this case a freezing behavior that is characterized by non-movement except for respiration. This modelled CR represents conditioned fear memory [1,2], reviewed in [3].

Conditioning of contextual or auditory cued fear underlies distinct mechanisms with numerous brain regions being involved [4-7]. For example, studies based on specific lesions in rodents provide evidence that the neural circuits involved in context and cued conditioning only partially overlap. Whereas both, hippocampus [8-10] and the amygdala seem to be involved in context-associated fear memory, the latter shows additional effects on cue conditioning [11,12].

Contextual fear long-term memory is reflected by a robust freezing behavior, often lasting several weeks or months [9,10]. This approach is highly amenable to investigate underlying neural circuitries which address either the acquisition phase or the ability to retrieve an already acquired memory by lesion of specific brain regions or pharmacological interventions at any time point during the experiment. In contrast, investigation of memory enhancement in non-lesioned animals is very time intensive with this model since the control group naturally forgets the learned association only months after acquisition. As a result, pharmacological interventions such as the NMDA receptor antagonist MK 801 or the GABA_A_ receptor agonist muscimol [13-15] have commonly been used to disrupt memory, thereby providing an option to study memory enhancement. However, these lesions bring caveats and limitations, rendering it a mechanistic model of reversal of impairments instead of necessarily studying memory enhancement for fear.

In this regard, it is of interest to adapt the CFC protocol such that loss of fear association occurs over a shorter time frame. Additionally, it is noteworthy that several studies provide evidence that contextual memory becomes less specific over time [10,16,17]. Only during the first weeks after acquisition are the context memories specific for the trained environment, a period during which the contextual memory is thought to be hippocampus dependent [4,9,18]. In this regard, if the hippocampal mechanisms of contextual long-term memory are to be investigated, it would be necessary to test during the first two weeks after training.

Based on reviewing the literature involving the CFC task, we decided to assess three major parameters. First, pre-exposure to the environment is crucial to associate the context with the subsequent shock [19,20]. Additional pre-exposure results in stronger formation of contextual memory but, more importantly, under certain circumstances can lead to false or generalization of contextual fear [21-23]. Second, in contrast to the “delay” protocol, the “trace” protocol separates the commencement of the US from the CS by an interstimulus interval (ISI) that can last up to 60 s. Third, the intensity of a single foot shock strongly influences the CR one day later [10,24]. However, investigations following repeated foot shocks of different intensities to evaluate the time dependence of forgetting have not been reported.

To address these parameters, we designed our experiments as follows. First, throughout the whole experiment we allowed the animals to pre-explore the chamber only once to avoid over-emphasis of the contextual stimuli; Second, to assess the influence of the ISI on both contextual as well as cue memory, we used a ‘trace’ protocol with an ISI of either 2 s or 30 s. An ISI shorter than 2 s has been suggested to turn the tone-cue into the main predictor for the US with only minor relevance of the contextual cues, whereas an ISI of 3 s or longer has been postulated to enhance the emphasis of the context to the CS/US pairing [5,25,26]; Finally, variability of the strength, duration and the number of foot shocks were investigated to address their influence on fear memory acquisition and retention.

By modifying these parameters, our goal was to identify a protocol where acquired fear memory was naturally forgotten within two weeks. Such a protocol would open new avenues to address memory enhancing mechanisms without the necessity of pharmacological interventions.

## MATERIAL AND METHODS

### Animals

All animal procedures were performed according to the institutional and European Union guideline (Directive 2010/63/EU) and were approved by the Ethical Committee of the responsible regional council (Tübingen, Germany). Adult male Wistar rats of 250–300 g were obtained from Janvier Labs, Le Genest-Saint-Isle, France. The animals were housed three or four per cage with *ad libitum* access to food and water, living under a 12/12 h light/dark cycle (lights on at 6 a.m.). The housing room was in close proximity to the experiment room, separated by one door, making acclimation to the experiment room over days less significant. The animals were tested cage-wise with 30 min acclimation between carefully transport of the cage to the experiment room and start of the experiment. All experiments were conducted during the light phase.

### Contextual and cued fear conditioning

A schematic representation of the experimental design can be seen in supplemental **Figure S1.** The animals were handled and habituated to the experimenter two days before the experiment started. At testing day, after acclimation the rats were placed into a sound attenuated chamber (MedAssociates Inc.) with house light on and white background noise (100 Hz, 65 db), provided by the built-in fan. At training day, a 5 min habituation phase was followed by 3 consecutive tone-cues for 15 s (1000 Hz, 80 db, 50 ms rising time). 2 s or 30 s after the end of each tone a foot shock of 0.3 or 1 mA for 0.5 or 2 s was delivered through a grid floor. The intervals between the first and second foot shock and between second and third shock were 124 s and 160 s, respectively. The freezing level of each animal was measured during each tone presentation. To gain control groups with high CR we used foot shocks of 1 mA for 2 s in all experiments. For the trials of acquisition as well as for contextual fear memory the chambers were cleaned with 70% ethanol, for cued fear memory experiments the chambers were cleaned with 1% acetic acid to provide a different odor. Additionally, a drop of ethanol or acetic acid was placed under the foot grid to ensure different olfactory sensation during the respective test.

Seven days after acquisition the animals were tested for contextual memory retention in the same chamber but without the presentation of the tone-cue. The animals were placed in the chambers for 5 min during which the freezing behavior of the animals were recorded (Video Freeze^®^ Version 2.5.5.0). One day later, the animals were tested for cued fear memory in chambers with different interior and odor but with the presentation of the tone-cue. In detail, the new interior consisted of a white plastic coverage on the foot grid to alter haptic sensation of the ground. The walls were covered with a white plastic coverage to alter geometry, visual and haptic sensation of the walls (from square and metal to round and white plastic). Olfactory sensation was altered as described above. The rats were placed in the chambers for 8 min during which the freezing behavior of the animals were recorded. After habituation for 2 min, serving as an indicator for generalized fear, the tone was presented for the last 6 min continuously. Data are shown as mean ± SEM. of the defensive response [% of total time]. The animal’s behavior was assessed by using linear detection method to percentage of freezing with a motion threshold of 18 arb. unit and a minimum freeze duration of 6 frames. Since the sample rate is fixed at 30 frames per second, motionless behavior must last at least 200 ms to be recognized as freezing.

### Statistical analysis

We used one-way analysis of variance (ANOVA) followed by Bonferroni’s post hoc test in case one factor (freezing level) was analyzed between three or more groups. In case of analyzing two factors (freezing level to each CS/US pairing) between three or more groups we used two-way ANOVA followed by Tukey’s post-hoc test. For comparison of only two groups, we used one-tailed *t*-test because in this case we anticipate the respective freezing level as being altered in just one direction. In all cases, values of *P* < 0.05 were considered to reflect statistically significant differences with ^*^P < 0.05, ^**^P < 0.01, ^***^P < 0.001, ****P < 0.0001.

## RESULTS

**Figure 1.**
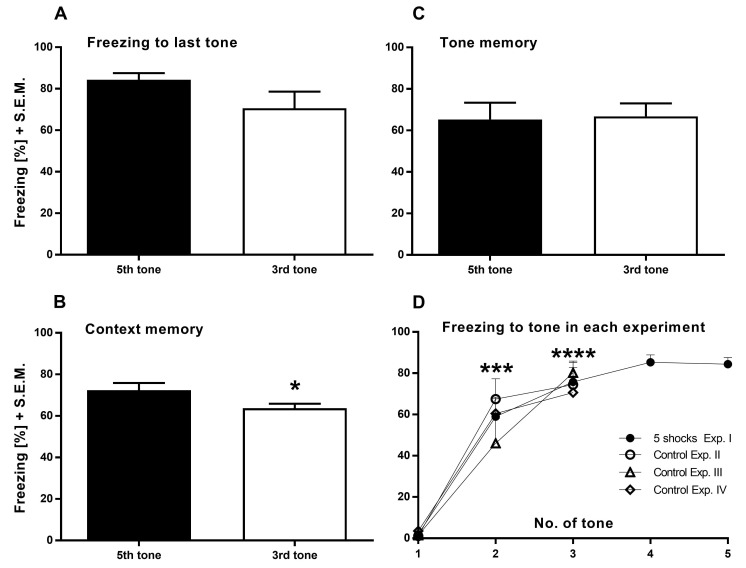
Experiment I. Three tone-cue/shock pairings revealed comparable freezing behavior to five tone-cue/shock pairings at the end of acquisition with slightly significant effect in contextual memory. **A.** Freezing to the 5th tone-cue (black bar) during acquisition revealed no significant difference to 3rd tone-cue (white bar). **B** and **C.** Compared with the five-pairs-group, the three-pairs-group froze significantly less during re-experience of the learned context seven days after acquisition (B) but not to the tone-cue (C) eight days after acquisition. **D.** Comparison between the five-pairs-group of experiment I (black circles, *N* = 8) and the controls of the following experiments II (empty circle, *N* = 8), III (triangle, *N* = 7) and IV (square, *N* = 8). All groups showed increased freezing to the second and third tone-cue compared with their respective first tone-cue, whereas the acquisition levels are comparable between the different experiments after the first three tone-cue presentations. Delivered foot shocks: 1 mA for 2 s after an ISI of 2 s. *P < 0.05, ***P < 0.001, ****P < 0.0001.

A prerequisite for adapting standard CFC protocol parameters was that the animals fully acquire the tone-cue/shock pairing. To initially determine the minimal number of shocks necessary to fully establish a fear associated memory, we investigated in an initial experiment I the acquisition by comparing groups of animals receiving a total of three or five shocks during the training phase. The freezing behavior of the animals was measured during each tone. This comparison revealed that there is no difference in memory acquisition after the second shock, *i.e.*, the level of freezing to the 3rd tone revealed no significant difference to the freezing during the 5th tone, suggesting comparable maximal associative learning with the second presentation of CS/US pairing. (**Fig. 1A**, unpaired one-tailed *t*-test; *P* = 0.0667). In addition, to determine if this fear memory was retained or not, the contextual and cued memory retention were subsequently measured 7 and 8 d, respectively, after training. The animals that received only 3 tone-cue/shock pairings froze slightly less during contextual memory trials (**Fig. 1B**; unpaired one-tailed *t*-test; *P* = 0.0268), indicating decreased retention after comparable acquisition. However, no difference in freezing was detected during tone presentation (**Fig. 1C**; unpaired one-tailed *t*-test; *P* = 0.4423).

To verify memory acquisition in the subsequently described experiments, **Figure 1D** compares the control groups of experiments II to IV and the five-shock group of this initial experiment I. All acquisition curves reflect learning since freezing levels to the second and third tone-cue (*i.e.*, after experiencing at least one foot shock) significantly differ from freezing to the first tone-cue (*i.e.*, before experiencing a foot shock; two-way ANOVA F_(2,81)_ = 108.4; *P* < 0.0001). This is supported by the fact that the CS/US association of each experiment was comparably acquired at the end of 3-shock training (two-way ANOVA; F_(3,27)_ = 0.1904, *P* = 0.9020).

**Figure 2.**
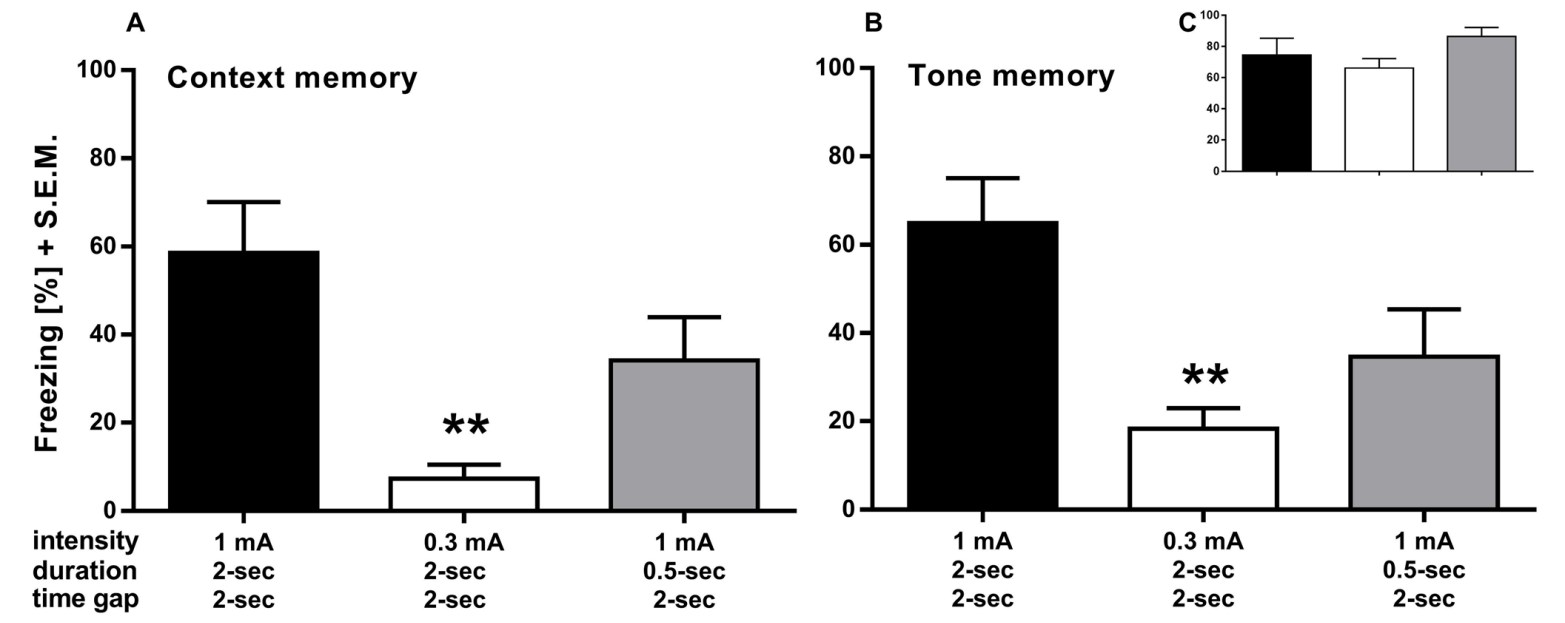
Experiment II. Contextual and cue memory is significantly decreased with delivered foot shocks of only 0.3 mA current strength during acquisition. **A** and **B.** The 0.3 mA group froze significantly less to the learned context seven days (A) and to the learned tone eight days (B) after acquisition. **C.** Freezing to the last tone during acquisition. Control group (black bar) received 3 foot shocks (1 mA for 2 s) after an ISI of 2 s between tone and shock whereas the other groups differed in the designated parameter. **P < 0.01.

Based on these initial findings, in the following experiments, we adapted different parameters compared to a control group that received 3 tone-cue/shock pairings of 2 s duration, 1 mA current strength and an ISI of 2 s, where only one parameter was changed in each group. **Figure 2** shows that delivering foot shocks of 0.3 mA significantly decreased memory for both context (**Fig. 2A**; one-way ANOVA; F_(2,21)_ = 8.176, *P* = 0.0024) and tone-cue (**Fig. 2B**; one-way ANOVA; F_(2,21)_ = 6.983, *P* = 0.0047) despite comparable freezing during the acquisition phase (**Fig. 2C**; one-way ANOVA; F_(2,21)_ = 1.665, *P* = 0.2132). In contrast, shortening the shock duration to 0.5 s did not reveal significantly decreased memory for context and tone-cue but only a trend. Since lowering the shock intensity to 0.3 mA still fully acquired the memory, yet showed decreased freezing to contextual re-experience, we used this group in the following experiments as a standard as well as to reproduce these findings.

**Figure 3.**
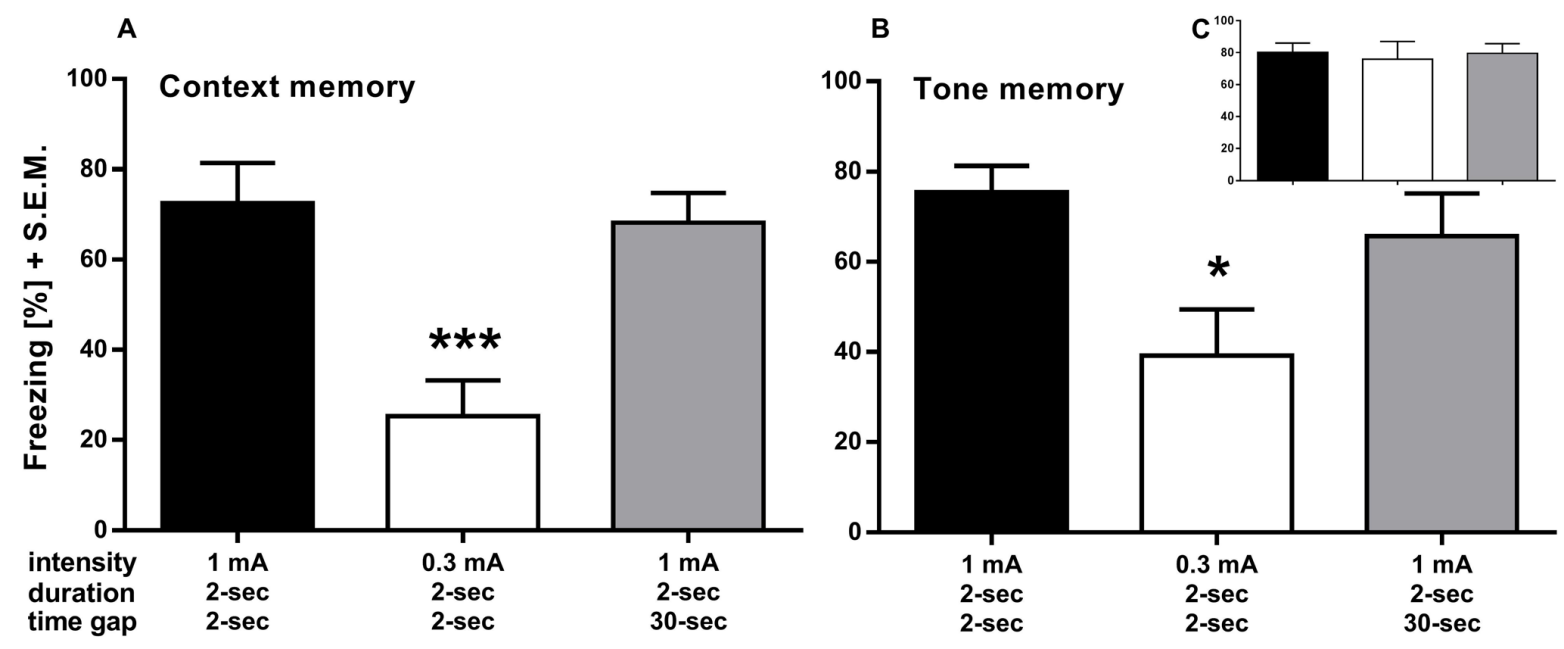
Experiment III. Contextual and cue memory is significantly decreased with delivered foot shocks of only 0.3 mA current strength during acquisition. **A** and **B.** The 0.3 mA but not the 30-s ISI group froze significantly less to the learned context seven days (A) and to the learned tone eight days (B) after acquisition. **C.** Freezing to the last tone during acquisition. Control group (black bar) received 3 foot shocks (1 mA for 2 s) after an ISI of 2 s between tone and shock whereas the other groups differed in the designated parameter. *P < 0.05, ***P < 0.001.

We subsequently addressed the ISI between tone-cue and shock, adapting it from 2 s to 30 s. Interestingly, **Figure 3** shows that ISI prolongation still acquired the memory equally well, yet did not reveal significant decreases in either the contextual or cued fear conditioning. Delivering foot shocks of 0.3 mA again significantly decreases memory for both context (**Fig. 3A**; one-way ANOVA; F_(2,18)_ = 11.10, *P* = 0.0007) and tone-cue (**Fig. 3B**; one-way ANOVA; F_(2,18)_ = 4.584, *P* = 0.0246) with comparable freezing to the last tone-cue during acquisition (**Fig. 3C**; one-way ANOVA; F_(2,18)_=0.08604, *P* = 0.9179).

**Figure 4.**
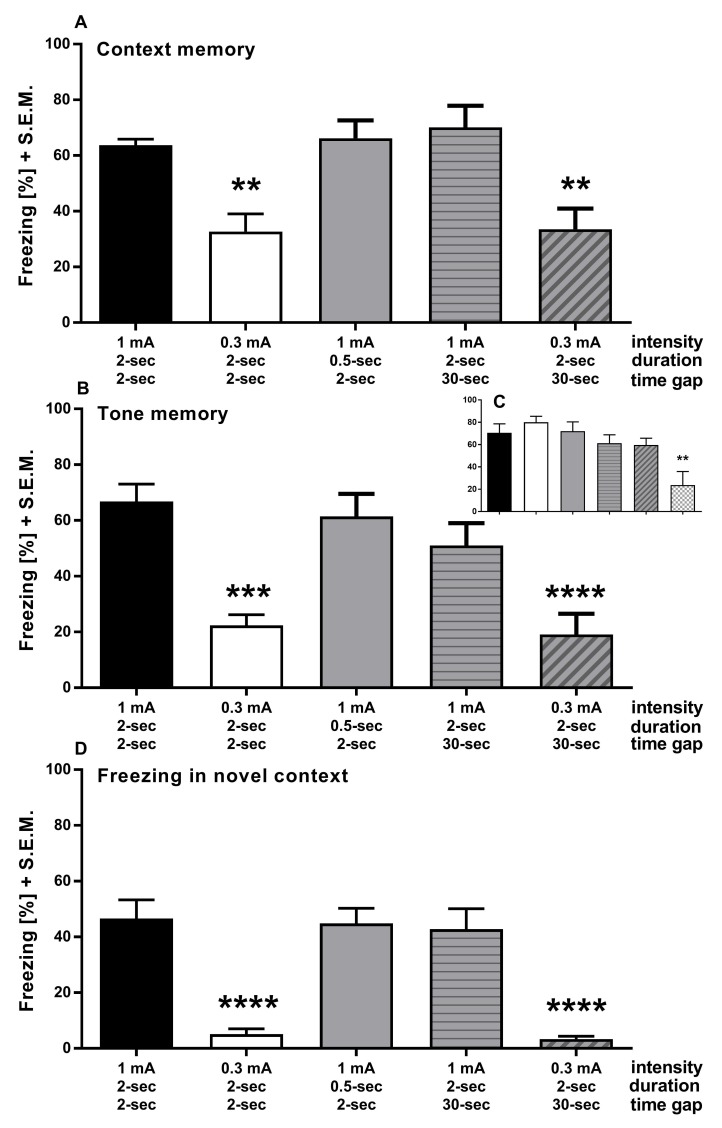
Experiment IV. Contextual and cue memory is not further decreased when ISI is extended before 0.3 mA foot shocks during acquisition. **A** and **B.** The 0.3 mA and the 0.3 mA + 30 s ISI group froze significantly less to both the learned context seven days (A) and the learned tone-cue eight days (B) after acquisition. **C.** Freezing to the last tone-cue during acquisition. Note the additional group, receiving a 0.3 mA foot shock for 0.5 s. This group was excluded from subsequent analysis due to weakly acquired association (last bar). All other groups showed comparable freezing levels. **D.** The weak foot shock of 0.3 mA significantly prevents freezing in a novel context with different odor and interior. Data were recorded during the two minutes habituation phase before testing for cue memory in (B). **P < 0.01, ***P < 0.001, ****P < 0.0001.

To assess if a combination of these factors might influence fear association memory, we addressed if an extended ISI or shorter shock duration with the 0.3 mA shock would significantly affect fear association. Therefore, new groups which combined either a 30 s ISI or a shock duration of 0.5 s with the current strength of 0.3 mA were additionally tested. All other groups (0.3 mA shock intensity, 0.5 s shock duration and 30 s ISI) were used as comparators to determine if the combined factors showed any additional influence or not. **Figure 4** shows that two groups delivering foot shocks of 0.3 mA showed significantly decreased memory for both context (**Fig. 4A**; one-way ANOVA; F_(4,35)_ = 8.543, *P* < 0.0001) and tone-cue (**Fig. 4B**; one-way ANOVA; F_(4,35)_ = 10.36, *P* < 0.0001). Interestingly, the ISI had no influence on the freezing level of these groups, indicating that the shock strength is the prevalent parameter that determines memory retention. Reproducibly, shortening the shock duration to 0.5 s or extending the ISI to 30 s in combination with a 1 mA shock intensity alone did not reveal a significant decrease in freezing behavior. All analyzed groups from this experiment showed a comparable level of learning during the training (**Fig. 4C**; one-way ANOVA; F_(4,35)_ = 1.372, *P* = 0.2637) with one exception: at the beginning of experiment IV one group receiving a 0.3 mA shock of 0.5 s duration did not acquire the memory as revealed by decreased freezing to the 3rd tone (**Fig. 4C**, last bar) and, thus, were excluded from analysis.

To determine if any of these protocols result in generalized fear expression in a novel context without a tone-cue, we measured the freezing level during the habituation time on day 8 before presenting the tone-cue (**Fig. 4D**). Compared to all conditions employing a 1 mA foot shock strength, the 0.3 mA groups showed significantly decreased freezing to the novel context (one-way ANOVA; F_(4,35)_ = 18.68, *P* < 0.0001). Together with the finding that the decreased freezing in the novel context is statistically comparable with the freezing level during habituation before acquisition at day 0 (**Fig. S3**; one-way ANOVA; F_(2,29)_ = 0.276, *P* = 0.7608), this indicates that the animals under such conditions did not show generalized fear.

**Figure 5.**
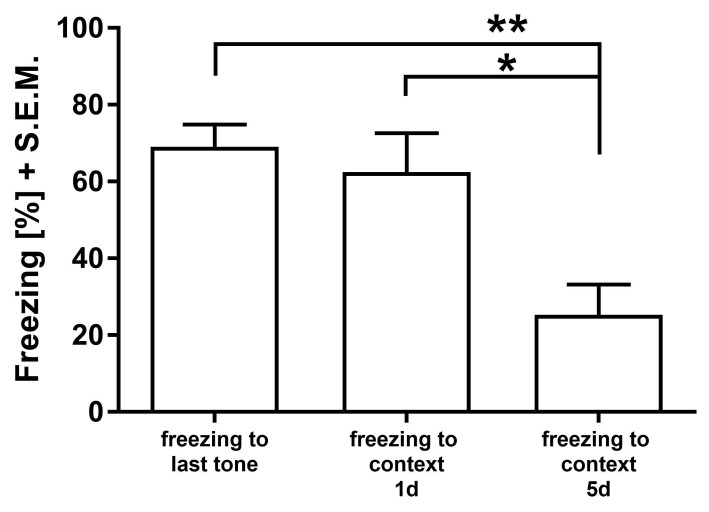
Freezing during acquisition is reflected in contextual memory in time-different manner. In a study of two distinct groups receiving three CS/US pairings with foot shocks of 0.3 mA memory for context was retained one day after training but forgotten 5 d after training. *P < 0.05, **P < 0.01.

During all experiments, the 0.3 mA protocol showed high freezing levels to the last tone-cue comparable to the strong protocol (**Fig. 1D**). To investigate if this cue-dependent acquisition level correlates with an established contextual fear memory, we additionally measured context-dependent fear at a shorter time-point after acquisition. For this purpose, we trained two groups of rats in the established “soft” protocol of three tone-cue/US pairings with a foot shock intensity of 0.3 mA after an ISI of 2 s. The context-dependent fear was then measured either one or five days after acquisition. In **Figure 5**, a similar level of freezing after the last tone-cue compared with contextual fear measured one day after the CS/US pairing was detected, confirming the acquisition of both cue and context memory. Importantly, context memory measured 5 d post acquisition was forgotten, as reflected in reduced freezing levels (one-way ANOVA, F_(2,25)_ = 11.45; *P* = 0.0003).

## DISCUSSION

Here we compared the impact of several parameters of the contextual and cued fear conditioning protocol on the capacity for rats to remember fear association. Our aim was to identify a protocol that allows natural forgetting within two weeks after contextual fear having been established.

### Acquisition

In all experiments, freezing of the control groups to the last tone-cue during acquisition was constantly measured between 70% and 80%, indicating a reliable and sufficient tone-cue/US pairing throughout our experiments. Intriguingly, the freezing response of the five-shock-group in experiment I reached a plateau after the third tone, indicating a saturated level of acquisition that cannot be further elevated. We interpreted these findings as a fully acquired pairing of the tone-cue/US association, especially since the freezing during the third tone-cue is a consequence of the experience of only the preceding two tone-cues/shock pairings and there is still an additional foot shock experience following. Moreover, since the animals demonstrated contextual fear one day after acquisition, the high level of freezing to the last tone-cue during acquisition correlates with contextual fear learning in this conditioning setup.

### Memory retention

Despite similar levels of freezing to both three and five shocks during the acquisition period, animals receiving three but not five shocks resulted in a slight, albeit significant reduction of contextual fear memory 7 d post acquisition. However, the animals still showed a strong fear memory, thereby complicating assessment of memory enhancing mechanisms. Therefore, in subsequent experiments, we used the three-shock protocol for the training as the control group.

Shock intensity: References [10] and [24] have shown increased shock intensity-dependent freezing or place avoidance behavior, where a low intensity foot shock did not show contextual fear memory one day after training in rats. However, both studies were limited to single shock training followed by a single memory test. This provides neither conclusions about the level of acquisition nor insight into time-dependency of forgetting contextual memory retention. Additionally, different shock intensities during acquisition have been used to assess memory extinction by repeatedly measuring the same animals over days [11]. Therefore, while this longitudinal study assessed certain consequences of using different shock intensities, they do not provide insight into natural forgetting in a time-dependent manner but rather shock intensity-dependent extinction of a learned association.

Lowering the intensity of the foot shocks from 1 mA to 0.3 mA during the acquisition period did not significantly reduce the acquisition of the fear association. However, it revealed a strongly decreased contextual memory one week after acquisition. This effect is quite robust since it was reliably decreased in all three experiments (II to IV) shown here. In contrast, neither shortening the duration of the foot shock to 0.5 s nor increasing the ISI to 30 s revealed any effect on behavioral freezing. These results indicate that the intensity of the electric shock is the main driver of emotional significance and that both shock duration and the time point of shock do not significantly influence the fear association. Indeed, combining the 0.3 mA foot shock with a 30 s ISI decreased neither context- nor cue-dependent freezing when compared with the 0.3 mA foot shock alone, further underscoring the impact of the shock intensity to the contextual and cued fear memory.

### Long trace conditioning

It has previously been demonstrated that a longer ISI results in a reduced tone-cued but increased contextual memory acquisition [27,28]. This suggests that the longer ISI uncouples the tone-cue from the US, leaving the contextual stimuli as the primary correlate to the US [29]. Conversely, substantial freezing to the tone-cue after long trace conditioning were reported by [26] and [5]. Therefore, it is interesting that we observed comparable freezing levels in the 2 s and 30 s ISI groups during both, training as well as in contextual and cued memory retention tests. It should be noted, however, that a potentially stronger freezing to the context might not be detectable due to an apparent ceiling effect on the level of freezing in our conditions. On the other hand, it is possible that, under certain conditions, the tone-cue—presented after a long ISI—is incorporated into the context as a contextual cue among many rather than being perceived as a distinct and direct predictor of the shock. Interestingly, merging the data of the 1 mA/30 s ISI groups from experiments III and IV revealed differences of extended ISI on cued or contextual memory, where cued memory was slightly, but significantly, reduced (**Fig. S2**). However, these effects are subtle, and a larger number of animals is necessary to differentiate these.

### Shock duration

Combining the constant shock intensity of 1 mA with a shock duration of either 2 s or 0.5 s, we noticed no impact of the shorter shock duration on freezing level to both context and tone-cue presentation during retention trials. However, animals that received a combination of the low intensity 0.3 mA shock with the short 0.5 s duration failed to acquire the tone-cue/US association during the training phase. We consider it likely that the lack of acquired tone-cue/US pairing also represents an insufficient pairing of context/US stimuli as well. Therefore, using this combination might not establish memory acquisition in the first place. Consequently, this constellation was not considered for subsequent analysis.

### Generalized fear

All groups receiving 1 mA shocks showed increased freezing in a novel chamber (without tone-cue) compared to the groups that were conditioned with 0.3 mA shocks, suggesting a certain level of generalized fear. This generalization does not, however, preclude an even higher level of freezing upon the presentation of a tone-cue (compare **Fig. 4B** and **Fig. 4D**). Generalization of contextual fear is measurable one or two weeks after acquisition [10,16,18]. Thus, beyond the possibility that the low shock intensity does not trigger generalization of fear at all, it is also conceivable that generalized fear has not yet manifested itself in the 0.3 mA groups.

In conclusion, our data suggest a significant impact of the shock intensity to associative memory formation, as opposed to the shock duration or a long ISI. Our initial experiments have shown decreased context memory 5 and 7 as well as cue memory 8 d after the acquisition of the tone-cue/US pairing in the low shock intensity group. Additionally, we demonstrated a comparable acquisition of contextual fear memory when the low shock intensity group was tested only 1 d post training, confirming that this soft protocol enables the animals to likewise acquire the contextual memory. Therefore, we have identified a protocol in which animals acquire both cue and context memory, but naturally forget this association in a short period of time. Indeed, we have successfully used this protocol to investigate the effect of hippocampal overexpression of Protein kinase Mζ on long-term contextual memory enhancement without pharmacological interventions [30]. Overall, our results suggest that this protocol allows exploration of memory enhancing mechanisms without the disadvantages of pharmacological interventions.

## Supplementary Material

Supplementary information **Figure S1.** Schematic representation of the experimental design.
**Figure S2.** Long Trace protocol decreased freezing to tone-cue.
**Figure S3.** Lack of fear generalization.
Supplementary information of this article can be found online at
http://www.jbmethods.org/jbm/rt/suppFiles/256.
